# Prognostic and clinicopathological significance of prognostic nutritional index (PNI) in patients with oral cancer: a meta-analysis

**DOI:** 10.18632/aging.204576

**Published:** 2023-03-07

**Authors:** Menglu Dai, Qijun Sun

**Affiliations:** 1 Clinical Laboratory, Huzhou Central Hospital, Affiliated Central Hospital of Huzhou University, Huzhou 313000, Zhejiang, China; 2 Stomatology Therapeutic Center, Huzhou Central Hospital, Affiliated Central Hospital of Huzhou University, Huzhou 313000, Zhejiang, China

**Keywords:** meta-analysis, prognostic nutritional index, oral cancer, survival, prognostic markers

## Abstract

Accumulating literature has explored how prognostically significant the prognostic nutritional index (PNI) was for the oral carcinoma population, but with inconsistent findings. Therefore, we retrieved the most recent data and carried out this meta-analysis to comprehensively analyze the prognostic performance of pretreatment PNI in oral cancer. The electronic databases of PubMed, Embase, China National Knowledge Infrastructure (CNKI), Cochrane Library and Web of Science were fully retrieved. PNI’s prognostic value for survival outcomes in oral carcinoma was assessed by estimating pooled hazard ratios (HRs) plus 95% confidence intervals (CIs). We examined the correlation of PNI with clinicopathological traits of oral carcinoma by utilizing the pooled odds ratios (ORs) plus 95% CIs. According to the pooled results of the present meta-analysis, which enrolled 10 studies involving 3,130 patients, for oral carcinoma suffers whose PNI was low, their disease-free survival (DFS) (HR=1.92, 95%CI=1.53-2.42, p<0.001) and overall survival (OS) (HR=2.44, 95%CI=1.45-4.12, p=0.001) would be inferior. Nonetheless, cancer-specific survival (CSS) was not linked significantly to PNI for the oral carcinoma population (HR=1.89, 95%CI=0.61-5.84, p=0.267). Significant associations of low PNI with TNM stages III-IV (OR=2.16, 95%CI=1.60-2.91, p<0.001) and age ≥ 65 years (OR=2.29, 95%CI=1.76-2.98, p<0.001) were found. As suggested by the present meta-analysis, a low PNI was linked to inferior DFS and OS among oral carcinoma patients. Oral cancer patients with low PNI may have high-risk of tumor progression. PNI could be served as a promising and effective index to predict prognosis in patients with oral cancer.

## INTRODUCTION

As one of the most universal malignancies of head and neck with poor prognosis and increasing incidence rate worldwide [1], oral carcinoma features distinct geographic disparity regarding its morbidity and prevalence across the world [2]. Its five-year rate of survival is around 63% [3]. The most frequently occurring cancer in the head and neck region is oral squamous cell carcinoma (OSCC), which represents 90% of entire oral carcinomas [4]. Management of oral cancer is based on surgical resection with or without adjuvant radiotherapy or chemoradiotherapy (CRT) [5]. Recent studies also showed that immunotherapy with immune checkpoint inhibitors (ICIs) also exhibited a marked tumor regression effect in some selected patients with OSCC [6, 7]. Although the treatment methods including ICIs have been applied, the prognosis of oral cancer is not substantially improved in the past several decades. Prediction of the prognosis is of major importance in oral cancer and biomarkers can provide guides to form personally optimized treatments of the disease [8]. Therefore, detection and identification of reliable and cost-effective biomarkers is urgently needed for oral cancer.

Parameters derived from the peripheral blood are important sources of biomarkers for oral cancer, including ratio of neutrophils to lymphocytes [9], ratio of platelets to lymphocytes [10], ratio of lymphocytes to monocytes [11], as well as prognostic nutritional index (PNI) [12, 13]. Also called Onodera’s PNI, the PNI indicator is computed from the overall quantity of peripheral blood lymphocytes and serum albumin [14]. PNI is calculated on the formula: 10 × serum level of albumin (g/dl) + 0.005 × peripheral blood quantity (per mm^3^) of lymphocytes. PNI is capable of reflecting host’s immune and trophic statuses. Previous studies have reported that low PNI played a prognostic role in diverse carcinomas, such as esophageal squamous cell carcinomas [15], gastrointestinal stromal tumors [16], hepatocellular carcinoma [17], non-small cell lung cancer [18], and glioma [19]. Many studies [12, 13, 20–27] have investigated PNI’s prognostic significance to oral cancer prognosis as well. However, the results were not consistent. Therefore, to systemically and comprehensively investigate how prognostically significant PNI was in oral carcinoma, we conducted the present meta-analysis based on the latest retrieved data.

## RESULTS

### Literature search process

**Figure 1:**
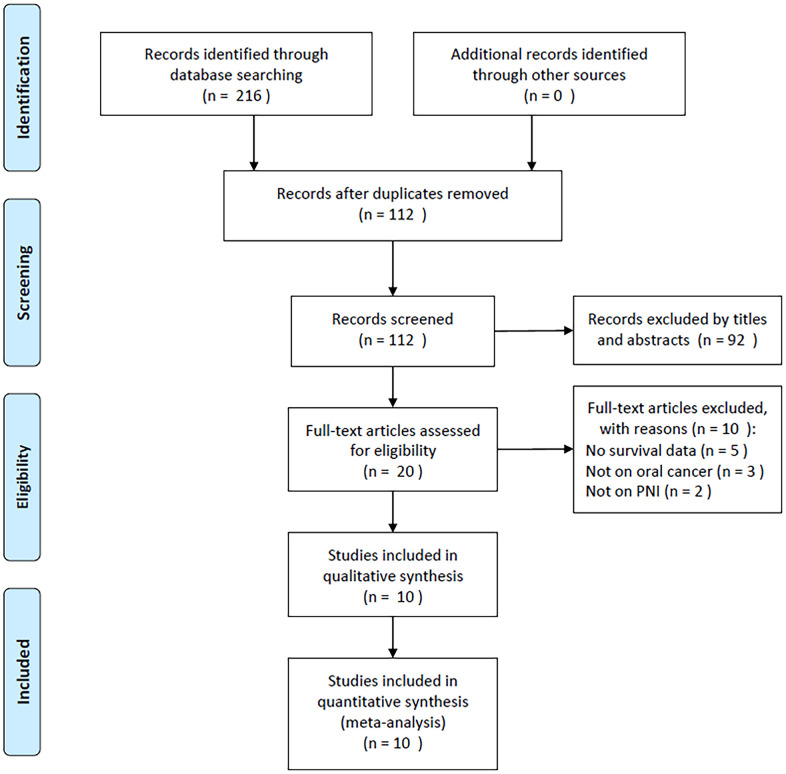
PRISMA flow chart showing selection of articles for review.

As shown in Figure 1, the number of records identified by initial literature retrieval totaled 216 and following elimination of duplicate items, 112 studies were retained for subsequent examination. Through abstract and title screening, ninety-two articles were excluded, while the remaining 20 studies were evaluated via full-text reading. Subsequently, 10 of them were discarded due to the fact that no survival data provided (n=5), not on oral cancer (n=3), and not reported on PNI (n=2). Finally, ten studies with 3,130 patients [12, 13, 20–27] were enrolled in the present meta-analysis (Figure 1).

### Characteristics of included studies

Table 1 demonstrates the baseline traits for the enrolled studies [12, 13, 20–27]. The years of publication ranged between 2020–2022 for these studies. Five studies were conducted in Japan [20–22, 25, 27], four in China [12, 13, 23, 26], and one in Taiwan [24]. Eight studies were published in English language [12, 13, 20–22, 24, 25, 27] and two in Chinese [23, 26]. All studies [12, 13, 20–27] were of retrospective design. Overall sample size was 3,130, varying between 47–1,395. Eight studies included patients with OSCC [13, 20–26], whereas 2 studies recruited oral cancer patients [12, 27]. Eight studies were conducted in single center [12, 20–24, 26, 27] and two studies were multicenter studies [13, 25]. The threshold scope for PNI was 42.685–52.44, with a median of 48.9. Threshold determination was accomplished based on the receiver operating characteristic (ROC) curve in 8 studies [12, 20–26]. Meanwhile, one study used the X-tile software [13], and one study referred to literature [27]. Nine studies reported PNI’s role in prognosticating OS outcome [12, 13, 20, 22–27], six studies provided the data of PNI for DFS prognosis [13, 20, 22, 24, 25, 27], and 2 works reported PNI’s correlation with CSS [20, 21]. The enrolled studies had NOS scores varying between 7–9, and the median value was 8, which indicated that all included studies were of high quality. In the subgroup analysis shown below, PNI cut-off value = 49 and sample size =150 were used to divide into two group. These two values were selected in according to the median value of PNI and sample size. These two values are close to the median value in each category.

**Table 1:** The baseline characteristics of the included studies.

**Study**	**Year**	**Country/region**	**Sample size**	**Age (year) Median (range)**	**Sex (M/F)**	**Cancer type**	**Clinical stage**	**Study period**	**Study center**	**Treatment**	**PNI cut-off value**	**Cut-off determination**	**Survival endpoints**	**Survival analysis**	**NOS score**
Bao, X.	2020	China	1,395	57.23 (20-80)	878/517	OC	I-IV	2007-2018	Single center	Mixed	49.3	ROC curve	OS	Multivariate	7
Wu, X.	2020	China	333	≤60: 133 >60: 200	175/158	OSCC	I-IV	2011-2018	Multicenter	Surgery	47.4	X-tile software	OS, DFS	Multivariate	8
Yoshida, R.	2020	Japan	47	79 (45-90)	23/24	OSCC	III-IV	2004-2011	Single center	CRT	42.685	ROC curve	OS, DFS, CSS	Multivariate	8
Yoshimura, T.	2020	Japan	103	68 (59-77)	61/42	OSCC	I-IV	2009-2015	Single center	Surgery	50.8	ROC curve	CSS	Multivariate	8
Abe, A.	2021	Japan	102	65.6	73/29	OSCC	I-IV	2008-2019	Single center	Surgery	42.9	ROC curve	OS, DFS	Multivariate	7
Duan, F.	2021	China	60	62.23	36/24	OSCC	I-IV	2019-2021	Single center	Surgery	48.5	ROC curve	OS	Multivariate	7
Fang, K. H.	2021	Taiwan	360	59 (31-88)	325/35	OSCC	I-IV	2007-2017	Single center	Surgery	51.75	ROC curve	OS, DFS	Multivariate	9
Watabe, Y.	2021	Japan	110	68	61/49	OSCC	I-IV	2004-2012	Multicenter	Surgery	52.44	ROC curve	OS, DFS	Multivariate	8
Xia, X.	2021	China	437	61.5 (21-83)	289/148	OSCC	I-IV	2015-2017	Single center	Surgery	46.23	ROC curve	OS	Univariate	7
Kubota, K.	2022	Japan	183	66 (26-93)	103/80	OC	I-IV	2005-2017	Single center	Mixed	52.44	Literature	OS, DFS	Multivariate	8

OC, oral cancer; OSCC, oral squamous cell carcinoma; ROC, receiver operating characteristic; OS, overall survival; DFS, disease-free survival; NOS, Newcastle-Ottawa Scale; CSS, cancer-specific survival; M, male; F, female; CRT, chemoradiotherapy.

### Prognostic role of PNI for OS in oral cancer

**Figure 2:**
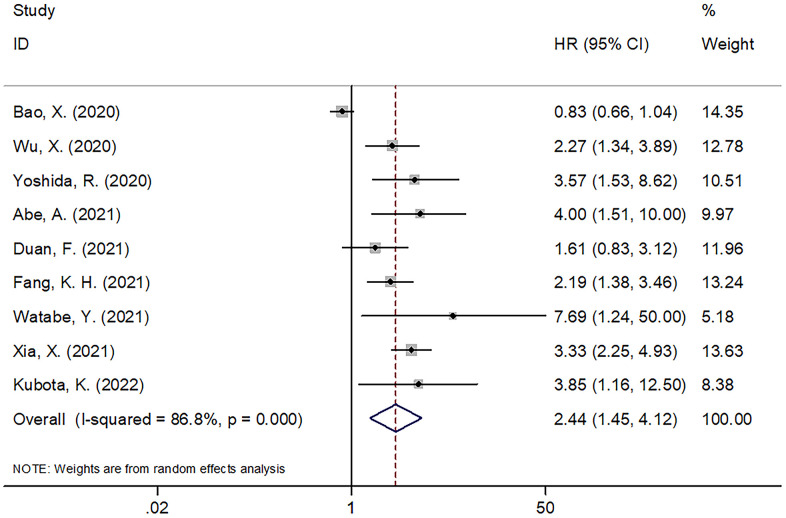
The forest plot of the association between PNI and OS in patients with oral cancer.

PNI was reported to be prognostically significant for OS of oral cancer in 9 studies involving 3,027 patients [12, 13, 20, 22–27]. We adopted a random-effects model since the heterogeneity was significant (I^2^=86.8%, Ph=0.000; Table 2 and Figure 2). As indicated by the pooled results of HR=2.44, 95%CI=1.45–4.12, p=0.001, a low PNI was linked significantly to inferior OS in oral cancer. We further performed subgroup analysis, as shown in Table 2, a low PNI was still a significant OS biomarker independent of study center, sample size, TNM stage, type of survival analysis, cut-off value, or method for threshold identification.

**Table 2:** Subgroup analysis of PNI for OS in patients with oral cancer.

**Subgroup factors**	**No. of studies**	**No. of patients**	**Effect model**	**HR (95%CI)**	**p**	**Heterogeneity**	
						**I^2^(%)**	**Ph**
Total	9	3,027	Random	2.44(1.45-4.12)	0.001	86.8	0.000
Geographical regions							
Japan	4	442	Fixed	4.01(2.34-6.87)	<0.001	0	0.908
China	4	2,225	Random	1.77(0.81-3.82)	0.150	92.8	<0.001
Taiwan	1	360	-	2.19(1.38-3.47)	0.001	-	-
Sample size							
≤150	4	319	Fixed	2.66(1.71-4.15)	<0.001	35.4	0.200
>150	5	2,708	Random	2.09(1.05-4.19)	0.037	91.8	<0.001
Cancer type							
OSCC	7	1,449	Fixed	2.66(2.12-3.33)	<0.001	13.8	0.324
OC	2	1,578	Random	1.59(0.36-7.04)	0.541	83.9	0.013
Study center							
Multicenter	2	443	Fixed	2.49(1.49-4.17)	0.001	35.3	0.214
Single center	7	2,584	Random	2.31(1.26-4.23)	0.007	89.1	<0.001
TNM stage							
I-IV	8	2,980	Random	2.34(1.34-4.07)	0.003	87.6	<0.001
III-IV	1	47	-	3.57(1.50-8.47)	0.004	-	-
Treatment							
Surgery	6	1,402	Fixed	2.60(2.06-3.29)	<0.001	23.0	0.261
Mixed	2	1,578	Random	1.59(0.36-7.04)	0.541	83.9	0.013
CRT	1	47	-	3.57(1.50-8.47)	0.004	-	-
PNI cut-off value							
≤49	5	979	Fixed	2.77(2.14-3.60)	<0.001	18.5	0.297
>49	4	2,048	Random	2.10(0.88-4.99)	0.094	86.9	<0.001
Cut-off determination							
ROC curve	7	2,511	Random	2.38(1.27-4.45)	0.007	89.2	<0.001
X-tile/literature	2	516	Fixed	2.48(1.52-4.04)	<0.001	0	0.426
Survival analysis							
Multivariate	8	2,590	Random	1.30(1.10-1.55)	0.003	83.3	<0.001
Univariate	1	437	-	3.33(2.25-4.93)	<0.001	-	-

OC, oral cancer; OSCC, oral squamous cell carcinoma; ROC, receiver operating characteristic; OS, overall survival; CRT, chemoradiotherapy.

### Prognostic significance of PNI for DFS and CSS in oral cancer

**Figure 3:**
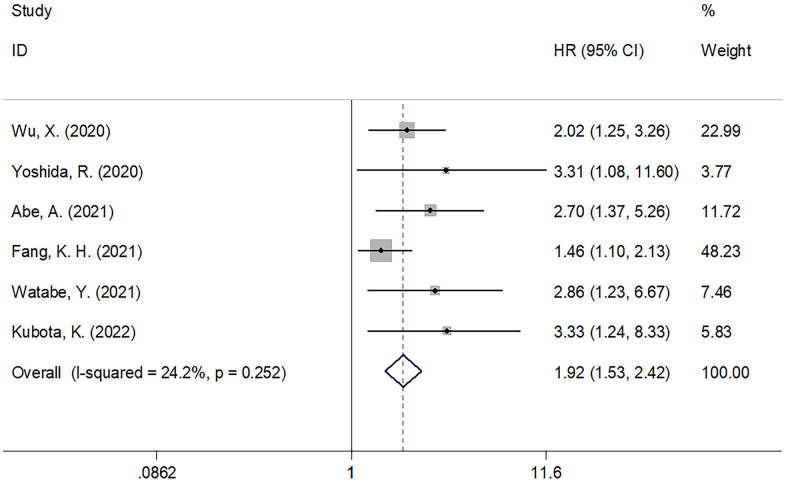
The forest plot of the association between PNI and DFS in patients with oral cancer.

**Figure 4:**
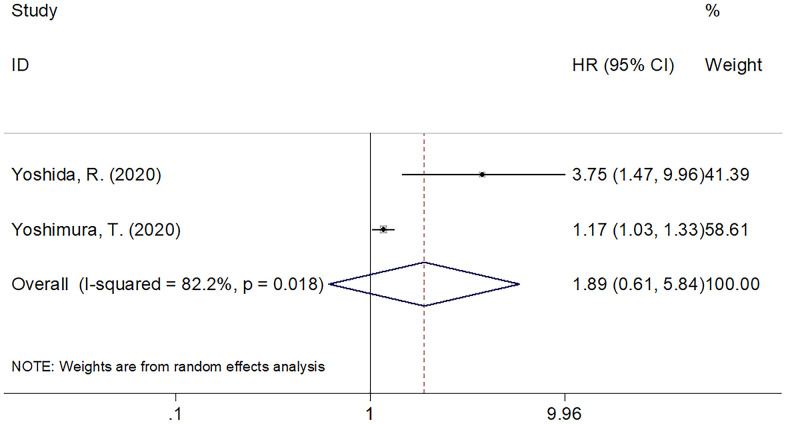
The forest plot of the association between PNI and CSS in patients with oral cancer.

Six studies with 1,135 patients [13, 20, 22, 24, 25, 27] provided the data on PNI and DFS for the oral carcinoma population. Since the heterogeneity (I^2^=24.2, Ph=0.252) was insignificant, we chose a fixed-effects model. As displayed in Table 3 plus Figure 3, the pooled HR was 1.92, while the 95% CIs were 1.53–2.42 (p<0.001), suggesting that a low PNI was connected to shortened DFS in oral cancer. As implied by subgroup analysis, a low PNI was still a significant DFS predictor in various subgroups of sample size, cancer type, study center, geographical locations, TNM stage, treatment method, threshold and method for identifying threshold (Table 3). Two studies including 150 patients [20, 21] analyzed PNI’s prognostic value for CSS. According to Figure 4, the pooled data revealed insignificant correlation of PNI with CSS in oral carcinoma (HR=1.89, 95%CI=0.61–5.84, p=0.267).

**Table 3:** Subgroup analysis of PNI for DFS and CSS in patients with oral cancer.

**Subgroup factors**	**No. of studies**	**No. of patients**	**Effect model**	**HR (95%CI)**	**p**	**Heterogeneity**	
						**I^2^(%)**	**Ph**
Total	6	1,135	Fixed	1.92(1.53-2.42)	<0.001	24.2	0.252
Geographical regions							
Japan	4	442	Fixed	2.94(1.91-4.51)	<0.001	0	0.983
China	1	333	-	2.02(1.25-3.26)	0.004	-	-
Taiwan	1	360	-	1.46(1.05-2.03)	0.026	-	-
Sample size							
≤150	3	259	Fixed	2.84(1.76-4.60)	<0.001	0	0.959
>150	3	876	Fixed	1.71(1.32-2.22)	<0.001	37.5	0.202
Cancer type							
OSCC	5	952	Fixed	1.86(1.47-2.36)	<0.001	23.8	0.263
OC	1	183	-	3.33(1.28-8.65)	0.014	-	-
Study center							
Multicenter	2	443	Fixed	2.20(1.45-3.33)	<0.001	0	0.481
Single center	4	692	Fixed	1.81(1.38-2.39)	<0.001	45.9	0.136
TNM stage							
I-IV	5	1,088	Fixed	1.88(1.49-2.38)	<0.001	30.6	0.217
III-IV	1	47	-	3.31(1.01-10.84)	0.048	-	-
Treatment							
Surgery	4	905	Fixed	1.81(1.42-2.31)	<0.001	30.3	0.231
Mixed	1	183	-	3.33(1.28-8.65)	0.014	-	-
CRT	1	47	-	3.31(1.01-10.84)	0.048	-	-
PNI cut-off value							
≤49	3	482	Fixed	2.31(1.60-3.35)	<0.001	0	0.647
>49	3	653	Random	2.10(1.19-3.70)	0.011	52.1	0.124
Cut-off determination							
ROC curve	4	619	Fixed	1.81(1.38-2.38)	<0.001	41.2	0.164
X-tile/literature	2	516	Fixed	2.23(1.45-3.43)	<0.001	0	0.357

OC, oral cancer; OSCC, oral squamous cell carcinoma; ROC, receiver operating characteristic; DFS, disease-free survival; CRT, chemoradiotherapy.

### The correlation between PNI and clinicopathological factors in oral cancer

**Figure 5:**
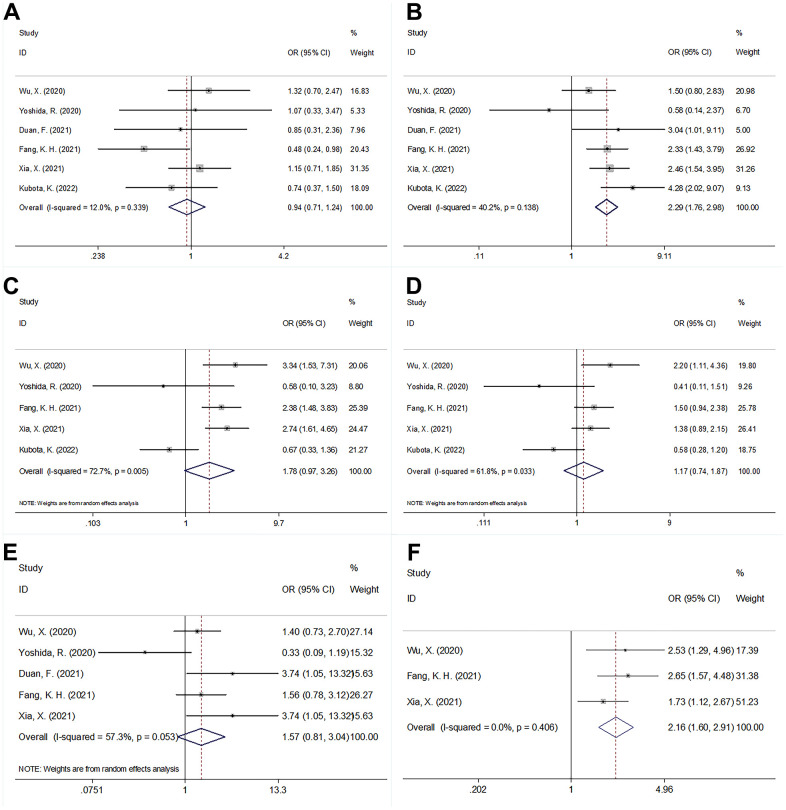
**The forest plots of the correlation between PNI and clinicopathological factors in patients with oral cancer.** (**A**) Sex (male vs female); (**B**) Age (years) (≥65 vs <65); (**C**) T stage (T3-4 vs T1-2); (**D**) N stage (N+ vs N0); (**E**) Differentiation (Poor, moderate vs well); and (**F**) TNM stage (III-IV vs I-II).

Data of PNI and clinicopathological traits in oral cancer were provided by 6 studies involving 1,420 patients in total [13, 20, 23, 24, 26, 27]. PNI’s correlations with the following parameters were examined: gender (male vs. female), age (years; ≥65 vs. <65), TNM stage (III–IV vs. I–II), N stage (N+ vs. N0), T stage (T3-4 vs. T1-2) and differentiation (Poor, moderate vs well). As suggested by the pooled data in Figure 5 combined with Table 4, a low PNI was linked significantly to age ≥ 65 years (OR=2.29, 95%CI=1.76-2.98, p<0.001) and III-IV TNM stage (OR=2.16, 95%CI=1.60-2.91, p<0.001). Nevertheless, the correlations of PNI with gender (OR=0.94, 95%CI=0.71-1.24, p=0.656), T stage (OR=1.78, 95%CI=0.97-3.26, p=0.064), N stage (OR=1.17, 95%CI=0.74-1.87, p=0.498), or differentiation (OR=1.57, 95%CI=0.81-3.04, p=0.184) were insignificant (Table 4 and Figure 5).

**Table 4:** The correlation between PNI and clinicopathological features in patients with oral cancer.

**Variables**	**No. of studies**	**No. of patients**	**Effects model**	**OR (95%CI)**	**p**	**Heterogeneity**	
						**I^2^(%)**	**Ph**
Sex (male vs female)	6	1,420	Fixed	0.94(0.71-1.24)	0.656	12	0.339
Age (years) (≥65 vs <65)	6	1,420	Fixed	2.29(1.76-2.98)	<0.001	40.2	0.138
T stage (T3-4 vs T1-2)	5	1,360	Random	1.78(0.97-3.26)	0.064	72.7	0.005
N stage (N+ vs N0)	5	1,360	Random	1.17(0.74-1.87)	0.498	61.8	0.033
Differentiation (Poor, moderate vs well)	5	1,237	Random	1.57(0.81-3.04)	0.184	57.3	0.053
TNM stage (III-IV vs I-II)	3	1,130	Fixed	2.16(1.60-2.91)	<0.001	0	0.406

### Publication bias

**Figure 6:**
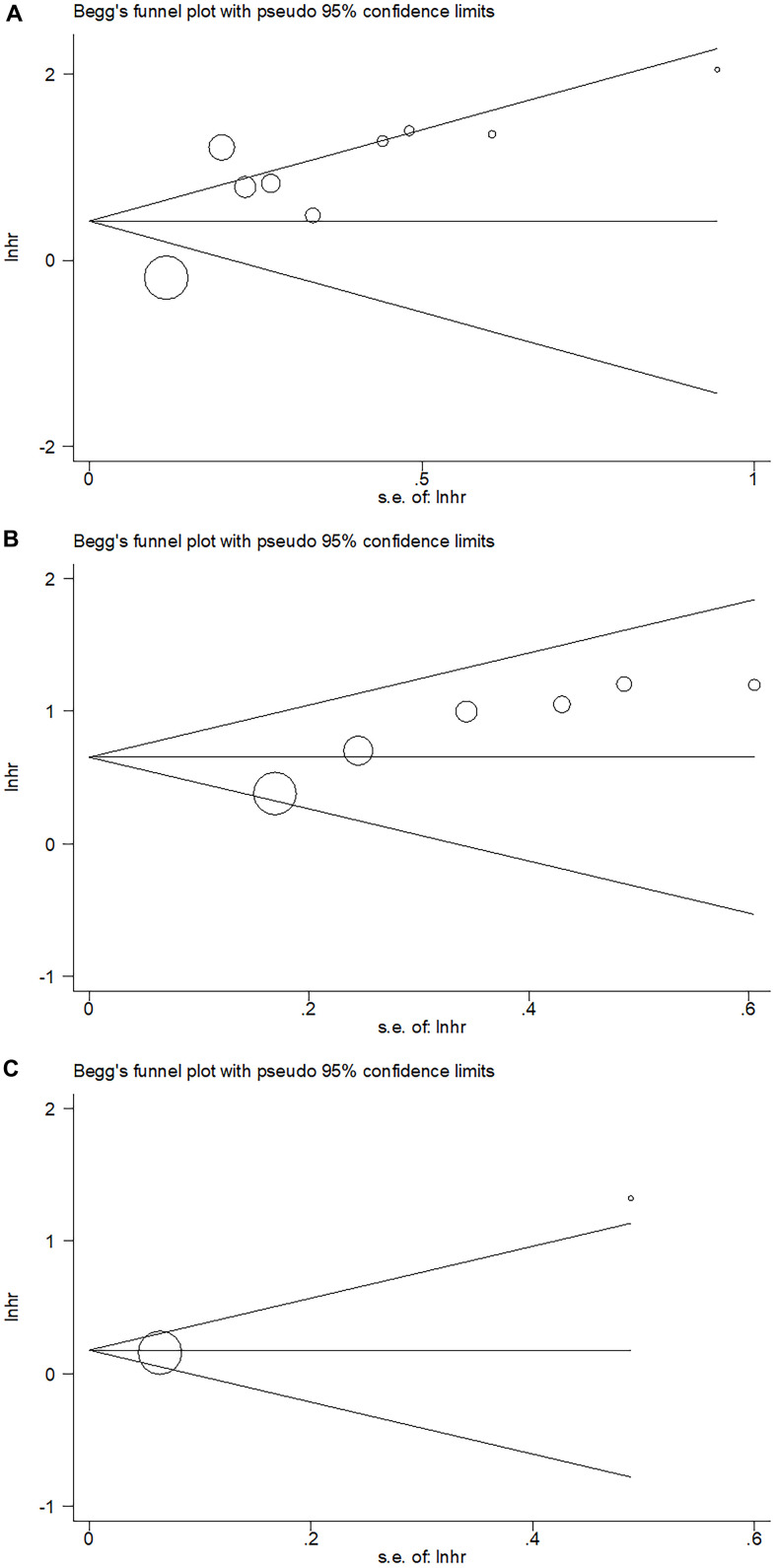
**Publication bias examined by Begg’s test.** (**A**) Publication bias for OS, Begg’s test: p=0.602; (**B**) Publication bias for DFS, Begg’s test: p=0.260; (**C**) Publication bias for CSS, Begg’s test: p=0.317.

Publication bias was assessed through Begg’s test combined with Funnel plotting. As shown in Figure 6, p values of Begg’s test for OS, DFS, and CSS were 0.602, 0.260, and 0.317, respectively. The funnel plots were symmetrical and the evidence of publication bias was absent in the current meta-analysis.

## DISCUSSION

There was inconsistency between former works regarding PNI’s prognostic significance in the oral carcinoma population. In our present meta-analysis, PNI’s exact role in prognosticating oral carcinoma was explored based on data gathered from 10 studies with 3,130 patients. As demonstrated by the pooled results, a low PNI acted as a significant predictor for DFS and OS, but not for CSS among the oral carcinoma population. Besides, a low PNI was linked significantly also to advanced stage of TNM and ≥65 years of age, which suggested the indicator role of PNI for disease progression and senile patients. Taken together, PNI acted as a significant biomarker for prognosticating clinical outcomes of oral carcinoma patients. Given its readily availability and cost efficiency, PNI was promising for prognostication in oral cancer. As far as we know, the present meta-analysis represents the initial attempt to investigate PNI’s prognostic value for oral carcinoma patients.

PNI is calculated according to the lymphocyte counts and albumin levels, therefore, the low levels of lymphocytes and serum albumin could lead to a low PNI. PNI is capable of reflecting host’s immune and trophic statuses. On the one hand, lymphocytes, particularly tumor-infiltrating lymphocytes (TILs), can induce the apoptosis of tumor cells [28]. TILs are an important component of cellular immunity and can eliminate tumor cells through humoral immunity [29]. Hence, lymphopenia can lead to compromised cancer resistance resulting from the immune-response to carcinoma cells [30]. On the other hand, albumin is the most abundant plasma protein, accounting for about half of the total protein content [31]. As an ordinary biomarker of trophic status, low albumin levels are associated with chronic inflammation that triggers IL-1, TNF-α and other cytokine stimulation [32]. Low albumin level indicates a malnutrition status and often occurs in patients with oral cancer because food intake ability is impaired [33]. Therefore, the decrease of PNI reflects the decreased inhibition of inflammatory response and malnutrition, thus affecting prognosis for oral carcinoma patients.

In prior meta-analysis-based works, PNI’s prognostic value in diverse types of carcinomas has been investigated [34–37]. According to a report by Kim et al., lower PNI was a negative predictor linked to progression of carcinomas and inferior survival of renal cell carcinoma patients in a meta-analysis including 9 studies [38]. According to a meta-analysis involving 1,311 patients by Luan et al., low PNI was linked to inferior PFS and OS in diffuse large B-cell lymphoma [36]. A recent meta-analysis involving 3,118 patients indicated that among the esophageal cancer population, there were associations of lower PNI with unfavorable prognostic predictor and inferior prognosis [37]. Dai and colleagues showed in their meta-analysis that among ovarian cancer population, low preoperative PNI was linked to shorter PFS, OS, as well as inferior clinicopathological traits [39]. In the current meta-analysis, we identified the low PNI as a negative prognostic indicator among the oral carcinoma population, showing conformance to the findings in other carcinomas. Besides, correlation of a low PNI with senile patients was noted as well. Therefore, patients with oral cancer ≥ 65 years may be suffer from high risk of tumor progression. The current meta-analysis retrieved literature with no language restriction. Notably, we included studies with a comprehensive literature search. Finally, ten studies with 3,130 patients were included in this meta-analysis. The sample size is relatively large to draw effective results.

Our meta-analysis also has a few shortcomings. Firstly, all included studies are from Asian regions, therefore, our results may more applicable to Asian population. Further works should validate PNI’s prognostic significance among the non-Asian population. Secondly, the cut-off values of PNI were not standard and various PNI cut-off value was used. Although one study [27] adopted 52.44 referring to a previous study [25], the other studies [12, 13, 20–24, 26] used different cut-off values. Thirdly, high heterogeneity existed in some analyses, which may be because retrospective researches are inherent in nature. Therefore, large-scale clinical trials with patients of diverse ethnicities are still needed to validate our findings.

Conclusively, the present meta-analysis suggested the correlations of a low PNI with inferior DFS and OS among the oral carcinoma patients. Oral cancer patients with low PNI may have high-risk of tumor progression.

## MATERIALS AND METHODS

### Study guideline and literature search

The procedure of current meta-analysis followed the statement of Preferred Reporting Items for Systematic Reviews and Meta-Analyses (PRISMA) [40]. The following electronic databases were fully retrieved: Web of Science, China National Knowledge Infrastructure (CNKI), Cochrane Library, PubMed and Embase. The updating date for last retrieval of literature was April 26, 2022. The following key words were used to retrieve potential research: “oral cancer”, “oral carcinoma”, “oral squamous cell carcinoma”, “OSCC”, “Squamous cell carcinomas of the tongue”, “prognostic nutrition index”, and “PNI”. There was no restriction on publication language. We also checked the references of enrolled works for relevant studies.

### Inclusion and exclusion criteria

The inclusion criteria were determined in accordance with the populations, interventions, comparators, outcomes, and study designs (PICOS) guideline.

We formulated the inclusion criteria as follows: (1) P (populations): subjects were diagnosed with oral carcinoma by pathological or histological means; (2) I (interventions): oral cancer patients with the PNI value was evaluated before treatment; (3) C (comparators): a threshold for distinguishing between low/high PNI was identified and the patient groups were divided as low PNI compared with high PNI; (4) O (outcomes): association of PNI with survival outcomes was reported in oral cancer; hazards ratios (HRs) for survival prognoses were reported in text, plus their 95% confidence intervals (CIs), or adequate data were offered for their computation; (5) S (study design): cohort studies, including prospective and retrospective cohorts published in any language.

The exclusion criteria were: (1) reviews, case reports, letters and conference abstracts; (2) absence of extractable survival data; (3) animal studies.

### Data extraction and quality assessment

Data from qualified studies were extracted by 2 independent researchers (MD and QS) according to a prespecified protocol. Disputes were all addressed by negotiation to consensus. The extracted information included name of the first author, study country/region, year, sample size, age, carcinoma type, study duration, Tumor–Node–Metastasis (TNM) stage, study center, study design, treatment method, threshold for PNI, method for identifying threshold, survival endpoints, type of survival analysis, HRs, as well as 95% CIs. All survival outcomes can be extracted included but not limited to disease-free survival (DFS), overall survival (OS), as well as cancer-specific survival (CSS). Based on the Newcastle-Ottawa Scale (NOS), the quality of included studies was evaluated by the foregoing MD and QS [41]. Through negotiation, discrepancies in evaluation were addressed until arriving at a consensus. The full score of NOS is 9 and the quality of studies was considered high when the NOS score was ≥6 points.

### Statistical analysis

The statistical analyses were entirely accomplished with the aid of Stata Ver. 12.0 (Stata Corp., College Station, TX, USA). PNI’s value for survival prognosis in oral carcinoma was assessed by estimating pooled HRs plus 95% CIs. The inter-study heterogeneity was evaluated by the Q and Higgins I-squared statistics. P for heterogeneity >0.10 and I^2^ ≤ 50% identified lower heterogeneity, in which case a fixed-effect model was adopted. In other cases, we utilized a random-effects model. The prognostic value of PNI in different patient populations was examined through subgroup analysis. For assessment of PNI’s correlation with clinicopathological traits in oral carcinoma, pooled odds ratios (ORs) plus 95% CIs were utilized. Publication bias was evaluated through Begg’s test combined with Funnel plotting. A p value <0.05 (two-sided) indicated a statistically significant difference.

### Availability of data and materials

The original contributions presented in the study are included in the article/supplementary material. Further inquiries can be directed to the corresponding author.
